# Metabolic Profiling of Somatic Tissues from *Monochamus alternatus* (Coleoptera: Cerambycidae) Reveals Effects of Irradiation on Metabolism

**DOI:** 10.3390/ijms150610806

**Published:** 2014-06-16

**Authors:** Liangjian Qu, Lijuan Wang, Qinghua Wang, Yuzhu Wang, Yongan Zhang

**Affiliations:** 1 Key Laboratory of Forest Protection, State Forestry Administration, Research Institute of Forest Ecology, Environment and Protection, Chinese Academy of Forestry, Haidian District, Beijing 100091, China; E-Mails: qulj2001@caf.ac.cn (L.Q.); wqh633@163.com (Q.W.); yzwang64@126.com (Y.W.); 2 State Key Laboratory of Tree Genetics and Breeding, Research Institute of Forestry, Chinese Academy of Forestry, Haidian District, Beijing 100091, China; E-Mail: wlj307@caf.ac.cn

**Keywords:** *Monochamus alternatus*, somatic cells, radiation, metabolomics

## Abstract

A high-level of sexual sterility is of importance for the sterile insect technique (SIT). However, the use of high-dose-intensity gamma radiation to induce sterility has negative impacts not only on reproductive cells but also on somatic cells. In this study, we investigated the metabolite differences in somatic tissues between non-irradiated, 20-Gy-irradiated, and 40-Gy-irradiated male *Monochamus alternatus*, an important vector of the pathogenic nematode, *Bursaphelenchus xylophilus*, which kills Asian pines. The results showed that metabolite levels changed moderately in the 20-Gy samples but were markedly altered in the 40-Gy samples compared with the non-irradiated samples. Twenty-six and 53 metabolites were disturbed by 20-Gy and 40-Gy radiation, respectively. Thirty-six metabolites were found to be markedly altered in the 40-Gy samples but were not changed significantly in the 20-Gy samples. The comprehensive metabolomic disorders induced by 40-Gy radiation dysregulated six metabolic pathways involved in the life process. The findings presented in this manuscript will contribute to our knowledge of the characteristic metabolic changes associated with gamma-radiation-induced damage to somatic cells and will allow for better exploration of the SIT for the control of this target pest.

## 1. Introduction

The sterile insect technique (SIT) is a biology-based, environmentally friendly method that has been used to suppress and eradicate a number of insect pest species [1]. An SIT program involves rearing large numbers of the target species, exposing them to gamma rays to induce sexual sterility, releasing them into the target population, and allowing the mating of the released sterile males with wild females to prevent them from reproducing [2]. In general, dominant lethal mutations that lead to sexual sterility are of importance for the SIT program. To induce a sufficiently high rate of a dominant lethal mutation, high-dose radiation is often used in the SIT program. However, it is well known that the gamma radiation used to induce sterility also has negative impacts on somatic cells and leads to a lower mating performance [2]. Such lapses in mating competitiveness can increase costs and compromise the effectiveness of SIT programs that release sterile insects [1,3,4,5]. Thus, to increase the effectiveness of an eradication program, the balance between the sterility level and fertilization potential should be considered [1,6]. Recently, an increasing number of studies have shown that partially-sterilized insects are more effective than completely sterilized insects, especially when the population density of the pest insect species is high [7,8,9,10,11].

Pine Wilt disease, which is caused by the pine wood nematode *Bursaphelenchus xylophilus* (Steiner and Buhrer, 1934) Nickle 1970, is a serious threat to the susceptible pine forests of the world [12]. This disease has caused substantial ecological damage as well as enormous economic losses in Asia [13,14]. The Japanese pine sawyer *Monochamus alternatus* Hope (Coleoptera: Cerambycidae), which is an important forest wood-boring pest, has been proven to be the most important and efficient vector of *B*. *xylophilus* in Asia [12,15,16]. Controlling *M*. *alternatus* is an efficient way to diminish the diffusion of *B*. *xylophilus* and reduce the damage caused by Pine Wilt disease [14,16].

Currently, SIT eradication program for *M*. *alternatus* is being developed in China. In our previous studies, adults of *M*. *alternatus* 3 to 5 days after emergence were irradiated with 20 to 160 Gy of gamma radiation from a ^60^Co source, and the optimal radiation dose for complete sterilization was investigated by evaluating the mating capability, longevity, oviposition, and egg hatching of the mated beetles. The results revealed that the males were more sensitive to irradiation than the females, and 40 Gy was found to be a suitable dose to achieve full sterility of the male beetles [17]. In a subsequent study, however, we found that the male beetles irradiated with 40 Gy (a dose that results in full sterilization) exhibited a poorer mating performance than males irradiated with 20 Gy (a dose that results in partial sterilization) and non-irradiated males. If 40 Gy is used in the SIT program for *M*. *alternatus*, sterile males must be continuously released to negate the lapses in their mating competitiveness, which will increase the cost of SIT and complicate the progress of the eradication program. The partially-sterilized males irradiated with a dose of 20 Gy have almost the same mating performance as non-irradiated males [17,18]. Exploring the changes in biochemical composition of somatic cells in irradiated male beetles with these two different intensities will provide insight into current question about SIT *M*. *alternatus* performance.

Metabolites are important fundamental elements of biology, and much of the actual activity that occurs in a cell is at the metabolite level. Compared with functional genomics analyses, the analysis of metabolites can provide a closer glimpse into the phenotypic state of an organism because metabolites are downstream of both genes and proteins [19]. Metabolomics, which is the analysis of the total population of metabolites in a given biological sample, has been widely applied to uncover biomarkers and metabolic fingerprints in humans and plants [20,21,22,23,24,25]. Recently, metabolomics has been used to identify global metabolomic changes induced by ionizing radiation and to discover biomarkers of radiation exposure in mouse urine and midgut, rat urine, and human lung and gut [26,27,28,29,30]. Although it is well known that that radiation used in SIT for insects can lead to lapses in mating competitiveness which may be a result of metabolic abnormalities in somatic cells, scarce information exists on the global metabolomic changes induced by radiation in somatic tissues. In the present study, we investigated the comprehensive metabolite profiles of somatic tissues from 20-Gy (a dose that results in partial sterilization)-irradiated, 40-Gy (a dose that results in complete sterilization)-irradiated, and non-irradiated male *M*. *alternatus* to reveal the effects of these two levels of ionizing radiation on the metabolism of somatic cells in the insects and to provide information that may be useful to the development of SIT for the control of *M*. *alternatus*.

## 2. Results and Discussion

### 2.1. Results

#### 2.1.1. Metabolic Profiling of Untreated and Irradiated Samples by GC-TOF/MS

**Figure 1:**
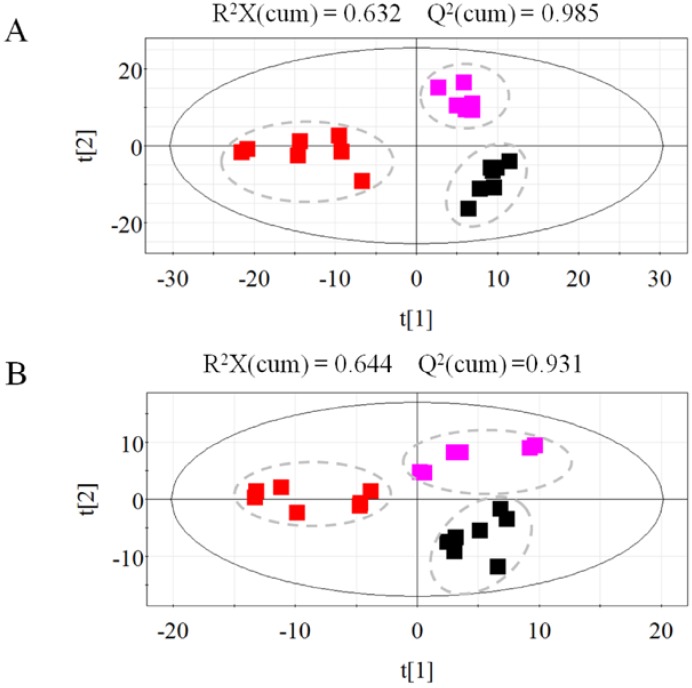
Score plots for the PLS-DA model of the lipophilic (**A**) and polar (**B**) profiles of non-irradiated, 20-Gy-irradiated, and 40-Gy-irradiated samples. The black squares represent the non-irradiated samples, the magenta squares represent the 20-Gy-irradiated samples, and the red squares represent the 40-Gy-irradiated samples.

To investigate the metabolite differences among the non-irradiated, 20-Gy-irradiated, and 40-Gy-irradiated beetles, the samples were analyzed using the GC-TOF/MS-based metabolomics technique. A total of 816 peaks were quantified, with 555 and 261 peaks identified in the lipophilic and polar phases, respectively. The chromatography data were mean-centered and scaled to pareto using SIMCA-P 11.5. PLS-DA was conducted to differentiate the samples based on the metabolic profiles. The model fit was evaluated using the R^2^ and Q^2^ parameters. The results showed that the PLS-DA models of the lipophilic and polar data had cumulative RX^2^, RY^2^, and Q^2^ values of 0.63, 0.99, and 0.98 and of 0.64, 0.98, and 0.93, respectively. Figure 1 shows the PLS-DA score plots derived from the lipophilic and polar profiles. The results showed that the non-irradiated, 20-Gy-irradiated, and 40-Gy-irradiated samples were separated by PLS components 1 and 2. In the lipophilic and polar score plots, the two PLS components accounted for 88% and 82% of the total variance, respectively. The 40-Gy-irradiated samples were separated from the non-irradiated and 20-Gy-irradiated samples along component 1 in the two plots.

#### 2.1.2. Metabolic Profiling of Untreated and Irradiated Samples by GC-TOF/MS

**Figure 2:**
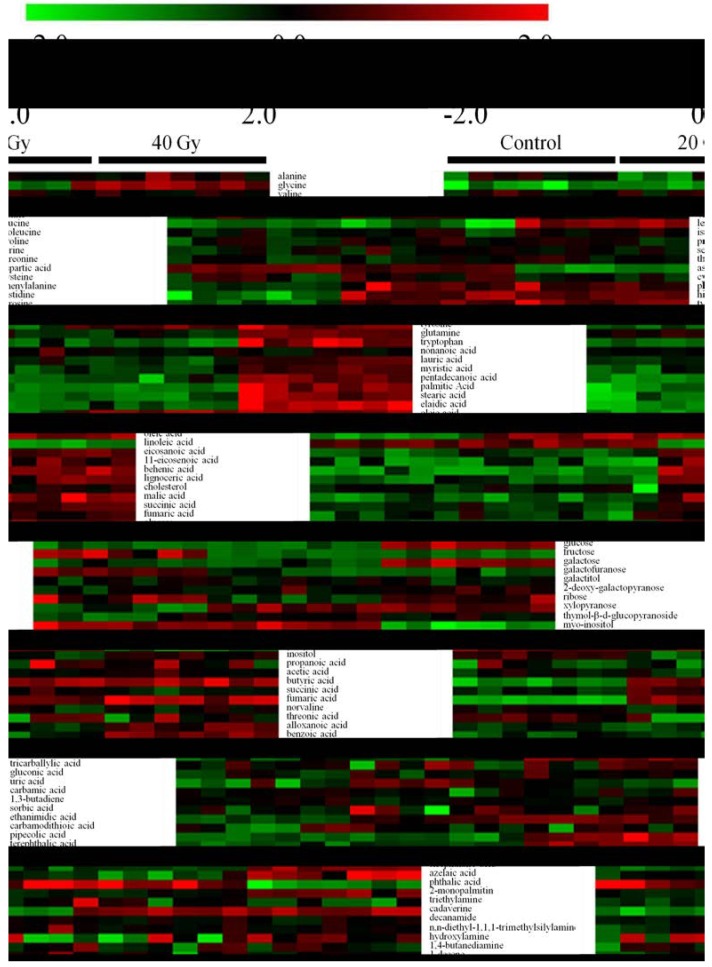
Heat map representation of the unsupervised hierarchical clustering of 98 metabolites across the non-irradiated and irradiated samples. The sample classes are indicated by the black bars (control = non-irradiated samples, 20 Gy = 20-Gy-irradiated samples, and 40 Gy = 40-Gy-irradiated samples). The columns represent the individual tissue samples, and the rows refer to distinct metabolites. Increases in the intensities of red and green indicate elevations and decreases, respectively, in the levels of a metabolite relative to the median metabolite expression.

Ninety-eight metabolites were identified by comparing the spectra of the samples against those from the commercial NIST library. A heat map of all of the identified metabolites is shown in Figure 2 to visualize the fluctuations among the non-irradiated and irradiated samples. As shown, the metabolite levels changed moderately in the 20-Gy samples and markedly in the 40-Gy samples compared with the non-irradiated samples. Twenty-six and 53 metabolites were disturbed by 20-Gy and 40-Gy radiation, respectively. Nine metabolites, namely phenylalanine, histidine, tyrosine, oleic acid, butyric acid, fumaric acid, ethanimidic acid, cadaverine, and octadecane, were significantly increased in both the 20-Gy- and the 40-Gy-irradiated samples. In addition to phenylalanine, histidine, and octadecane, all of the other metabolites presented greater alterations in the 40-Gy samples than in the 20-Gy samples. Aspartic acid, fructose, xylopyranose, 2,6-dimethyldecane, and dibutyl phthalate were decreased in both groups of irradiated samples, and the former three metabolites were decreased more by 40-Gy radiation than by 20-Gy irradiation. Three other metabolites showed a different trend: the levels of lauric acid and stearic acid were decreased in the 20-Gy-irradiated samples but increased in the 40-Gy-irradiated samples, and thymol-β-d-glucopyranoside showed the reverse trend. Eight metabolites were altered in the 20-Gy samples but not changed in the 40-Gy samples: the succinic acid, uric acid, phenelzine, and 6,13-octadecadien-1-ol acetate levels were increased, whereas the levels of ribose, inositol, undecane, and sitosterol were decreased. Additionally, 36 metabolites were found to be markedly altered in the 40-Gy samples but not changed significantly (*p* < 0.01) in the 20-Gy samples. These disturbed metabolites include six amino acids, nine fatty acids, five sugars, 11 organic acids, and five alkanes. Most of the detected amino acids, fatty acids, carbohydrates, and hydrocarbons were altered by 40-Gy radiation.

**Figure 3:**
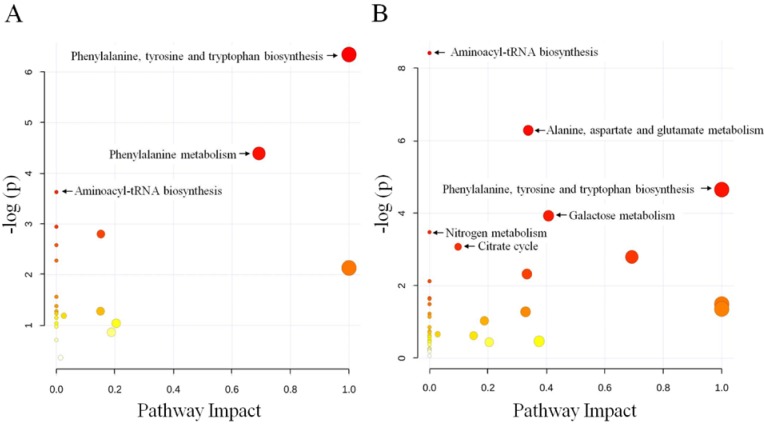
Schematic of the metabolome following the metabolite pathway mapping of the impacted metabolites identified after exposure to 20-Gy (**A**) and 40-Gy (**B**) radiation. The analysis was performed using the MetaboAnalyst software.

To determine whether our observations of changes in the metabolites did in fact reflect coordinate changes in defined metabolic pathways, we used MetaboAnalyst’s online pathway analysis to identify the network pathway. This analysis was based on the high-quality KEGG metabolic pathways as the back-end knowledgebase that helps researchers identify the most relevant pathways involved in the conditions under study. The detailed construction of the metabolism pathways with higher scores is shown in Figure 3, Tables S1 and S2. The results revealed that three pathways were significantly (*p* < 0.05) perturbed in the 20-Gy samples: phenylalanine, tyrosine, and tryptophan biosynthesis, phenylalanine metabolism, and aminoacyl-tRNA biosynthesis (Figure 3A). In contrast, six pathways were greatly (*p* < 0.05) influenced in the 40-Gy samples: aminoacyl-tRNA biosynthesis, alanine, aspartate, and glutamate metabolism, phenylalanine, tyrosine, and tryptophan biosynthesis, galactose metabolism, nitrogen metabolism, and citrate cycle (TCA cycle) (Figure 3B).

### 2.2. Discussion

Less competitive males that do not perform their intended function well will weaken the effectiveness of the SIT and alter the cost-to-benefit ratio of this strategy. In the present study, we investigated the metabolite profiles of the somatic tissues from 20-Gy-irradiated, 40-Gy-irradiated, and non-irradiated male *M*. *alternatus* to measure the metabolic abnormalities in the sterile males. Our examination revealed that the metabolite levels were changed slightly in the 20-Gy samples but markedly altered in the 40-Gy samples compared with the non-irradiated control.

The differential metabolites induced by radiation mainly included fatty acids, amino acids, carbohydrates, organic acids, and alkanes. Fatty acids are major energy reserves and important structural elements of the cell membranes of insects [31,32]. A study on *Ceratitis capitata* has shown that radiation exposure can cause alterations in the concentrations of saturated and unsaturated fatty acids [33]. In this study, fatty acids represented the largest group of differential metabolites. Two saturated fatty acids and one unsaturated fatty acid were altered in the 20-Gy-irradiated males, whereas eight saturated fatty acids and four unsaturated fatty acids were altered in the 40-Gy-irradiated males. The only detected polyunsaturated fatty acid (PUFAs) that was markedly decreased in the 40-Gy-irradiated males, *i*.*e*., linoleic acid, was not changed significantly in the 20-Gy-irradiated samples. Linoleic acid, an important PUFA in the cell membrane, determines the fluidity of the cell membrane [31]. The decreased level of linoleic acid due to irradiation may cause some pathological effects, including impairment of membrane functions, inactivation of membrane-bounded receptors, increased non-specific permeability to ions, and decreasing fluidity and motility [34]. Accordingly, the less fluid membranes will limit the flying ability and decrease the competiveness of the irradiated males [35]. Saturated and monounsaturated fatty acids, as components of phospholipids and di- and triglycerides, are also integral parts of cell membranes [31]. Perturbations in these fatty acids will make it difficult for the cell to conduct its normal functions and increase the cell’s susceptibility to injury and death. Microscopic examinations showed that radiation could damage the midgut tissues and cause inflammatory response in the intestines of the insects [36]. In this study, two inflammatory response-related fatty acids, namely eicosanoic acid and 11-eicosenoic acid [30,37], were indeed observed to be increased in the 40-Gy-irradiated males. Overall, the disturbance caused by 40-Gy radiation on the levels of fatty acids indicates malfunctions of the somatic cell membranes and may contribute to the poorer mating performance of the irradiated males.

Amino acids play important roles in insect development. In addition to their role in protein synthesis, they have additional functions related to the synthesis of phospholipids, detoxification, energy production, and neural transmission [38,39,40]. Several investigators have demonstrated that X- and Y-irradiation cause variations in the amounts of amino acids in organisms. For instance, in mice, radiation was observed to markedly alter the quantities of cysteine, cystine, glutamic acid, lysine, and phenylalanine [30,41]. In *C*. *capitata*, it has been reported that the radiation effect on the free amino acids involves an increase in the concentration of both individual amino acids and the total pool, and that methionine was the only amino acid that was found at a lower level in the irradiated group [42]. In the present study, the levels of four and 10 amino acids were altered in the 20-Gy- and 40-Gy-irradiated males, respectively. The four amino acids increased in the 20-Gy samples were further elevated by 40-Gy radiation. In the 40-Gy-irradiated males, nine amino acids were increased but only one amino acid, namely aspartic acid, was markedly decreased. Aspartic acid was found in two configurations in insects, and racemase can convert l-aspartic acid into d-aspartic acid. A previous study showed that d-aspartic acid can be markedly decreased by gamma radiation [30]. Thus, it was assumed that the decrease in the amount of aspartic acid found in this study may be derived from d-aspartic acid and not l-aspartic acid because the two configurations exhibit the same elution times through GC-TOF/MS. d-Aspartate has been proposed to act as a neurotransmitter in peripheral nerves. It acts via *N*-methyl-d-aspartate (NMDA) receptors that are present on the peripheral nerves and is involved in intestinal motility [39,40,43,44]. A decrease in d-aspartate induced by radiation may affect normal intestinal function through decreased peristalsis and digestive enzyme production [30]. Furthermore, decreased d-aspartate levels may suggest a lower activity of metabolic pathways, such as the TCA cycle, because aspartate is an endogenously produced amino acid [30]. Therefore, it is possible that the increased free amino acid concentration after irradiation may be attributed to a decreased capacity of the tissues to utilize the free amino acid pool, particularly with respect to protein synthesis.

In addition to fatty acids and amino acids, carbohydrates, TCA cycle intermediates, and hydrocarbons were also altered by radiation. The pathway enrichment analysis revealed that three pathways were perturbed in the 20-Gy samples, and six pathways were greatly influenced in the 40-Gy samples. Carbohydrate metabolism, nitrogen metabolism, and the citrate cycle, were disturbed in the 40-Gy-irradiated males but not influenced in the 20-Gy-irradiated samples. Carbohydrates are the major energy material at the start-up stage of insect flight and also play an important role in the structure and function of all of the tissues during insect life [45]. Nitrogen metabolism is one of the basic processes that forms and maintains insect life activity. The TCA cycle, an important pathway for the generation of energy, is the final pathway for the oxidation of carbohydrates, fatty acids, and amino acids. The disturbance of these three pathways in somatic tissue indicates disorders in the cell basal metabolism of the irradiated males. In this study, some hydrocarbons were also altered by irradiation but not mapped to a pathway. Hydrocarbons consisting of straight chain n-alkanes, methyl-branched alkanes, or unsaturated alkenes are the main components of surface waxes in insects. They provide the primary passive barrier to evaporative water loss and play a critical role in allowing insects to thrive in terrestrial environments [46]. Disturbed components of hydrocarbons in the surface waxes of insects would increase the transpiration of water through the cuticle and cause some pathological effect [47,48]. Furthermore, some hydrocarbons, such as heptacosane, are the components of the contact sex pheromone and are involved in the chemical communication associated with the mating behavior of insects. A strong correlation has been reported between the reproductive status of an individual and its hydrocarbon profile [49,50,51,52]. The serious disturbance in hydrocarbons observed in the 40-Gy-irradiated males may influence their mating behavior and decrease the effectiveness of the SIT.

## 3. Experimental Section

### 3.1. Insects and Irradiation

The larvae of *M*. *alternatus* were collected from pine logs in Hangzhou, Zhejiang, China, in March 2011, and reared on an artificial diet under constant temperature (25 ± 1 °C) with 65% relative humidity and 12-h light:12-h dark cycle conditions. Adult individuals four days after emergence were manually separated according to their sex, and the males were selected for the experiment. The males in almost the same body size were separated into three groups: one group was the non-irradiated control, and the other two groups were the 20-Gy-irradiation and 40-Gy-irradiation groups. Irradiation was performed at the Institute of Crops and Nuclear Technology Utilization of the Zhejiang Academy of Agricultural Sciences of China using gamma rays from a 60Co source at a dose rate of 1.0 Gy/min, and the final radiation doses were 40 and 20 Gy. Five days after radiation, seven samples from each group were randomly chosen for metabolite extraction.

### 3.2. Chemicals

Methanol and chloroform (HPLC grade; ≥99.9%) were purchased from Fisher Scientific (Hampton, NH, USA). Pyridine (GC-grade; ≥99.8%), *N*-methyl-*N*-(trimethylsilyl) trifluoroacetamide (MSTFA reagent), the methoxy amination reagent, and the internal standard reference compounds ribitol (≥99.0%) and nonadecanoic acid (≥99.0%) were purchased from Sigma-Aldrich (St. Louis, MO, USA). Distilled water was purified “in-house” using a Milli-Q system Millipore (Bedford, MA, USA).

### 3.3. Metabolite Extraction and Derivatization

Metabolite extraction was performed according to the protocol described in a previous study with some modifications [53]. The thoraxes and abdomens (testes were removed) of untreated and irradiated beetles were frozen with liquid nitrogen and ground to a powder with a mortar and pestle. Approximately 100 mg of the powder were used for the extraction of the metabolites. A volume of 1.5 mL of the extraction buffer (methanol/chloroform/water, 5:2:2), 10 µL of nonadecanoic acid (2.1 mg/mL), and 100 µL of ribitol (0.2 mg/mL) (internal quantitative standards) were added to an Eppendorf tube containing the frozen powder. The mixture was extracted using a supersonic instrument for 30 min and centrifuged at 11,000 rpm for 10 min. One milliliter of the supernatant was transferred to another tube, mixed with 300 µL of chloroform and 600 µL of dH_2_O, and centrifuged at 4000 rpm for 5 min. The upper polar phase (100 µL) and the lower lipophilic phase (100 µL) were dried under a nitrogen gas stream in a vacuum rotary evaporator without heating. The dried residues were oximated with 40 µL of methoxylamine hydrochloride (20 mg/mL) in anhydrous pyridine at 37 °C for 2 h and then silylated at 37 °C for 30 min with 70 µL of MSTFA. The derivatized samples were transferred to 250 µL glass vials (Agilent) for GC-TOF/MS analysis.

### 3.4. GC-TOF/MS

The polar and lipophilic phases were analyzed using a LECO Pegasus IV gas chromatography time-of-flight mass spectrometry (GC-TOF/MS) system. The GC-TOF/MS system was composed of an Agilent autosampler, a 6980 gas chromatograph (Agilent, San Jose, CA, USA), and a LECO Pegasus IV time-of-flight mass spectrometer (Leco, St. Joseph, MI, USA). One microliter of each derivatized sample was injected by an autosampler into the gas chromatograph, which was equipped with a 30 m × 0.25 mm i.d. fused silica capillary column with a chemically bonded 0.25-μm DB-5 MS stationary phase (J&W Scientific, Folsom, CA, USA). The injector temperature was 280 °C. The helium gas flow rate through the column was 1.5 mL/min. The column temperature was maintained at 80 °C for 4 min, increased at a rate of 5 °C/min to 330 °C, and maintained at 330 °C for 5 min. After this, the column effluent was introduced into the ion source of the mass spectrometer. The transfer line and the ion source temperatures were 250 and 200 °C, respectively. Ions were generated by a 70 eV electron beam at an ionization current of 2.0 mA, and 20 spectra were recorded in the mass range from 80 to 500 *m*/*z*. The acceleration voltage was turned on after a solvent delay of 300 s. The detector voltage was 1700 V.

### 3.5. Data Processing

The acquired MS files from the GC-TOF/MS analysis were exported in NetCDF format using the ChromaTOF software (v3.3, Leco, St. Joseph, MI, USA). The NetCDF files were processed by custom scripts in MATLAB (The Math Works, Natick, MA, USA) to perform the baseline correction, de-noising, smoothing, alignment, time-window splitting, and multivariate curve resolution [54,55,56]. The resulting three-dimensional matrix (referred to as the metabolic matrix hereafter) includes the sample information, peak retention time (RT), and peak intensities. The internal standards and any known artificial peaks, such as peaks caused by noise, column bleed, and the derivatization procedure, were removed from the matrix. The weight was normalized to the maximum for each sample to minimize the discrepancy resulting from different sample weights. Min-max normalization was then used to transform the data into the range of 0 to 1. The missing values were assumed to result from areas below the limits of detection. For each metabolite, the missing values were inputted with the observed minimum after the normalization step.

### 3.6. Univariate and Multivariate Statistical Analyses

The metabolic matrix was analyzed further using both univariate and multivariate statistical techniques in an attempt to evaluate the predictive power of each metabolite and to select potential differential metabolites. Non-parametric Mann-Whitney tests (SPSS17.0, IBM Corporation, Chicago, IL, USA) were the univariate methods selected for the differential metabolite evaluation and selection. A smaller *p* value obtained through the Mann-Whitney test indicated a greater significance of the corresponding metabolite. The critical *p* value was set to 0.01. In parallel, multivariate statistical analysis, unsupervised principle component analysis (PCA), partial least squares discriminant analysis (PLS-DA), and sophisticated supervised orthogonal projections to latent structures discriminant analysis (OPLS-DA) were conducted using the SIMCA-P 12.0 software package (Umetrics, Umeå, Sweden). The matrix was mean-centered and unit-variance-scaled prior to modeling. R^2^X and R^2^Y represented the fractions of the variance of the *x* and *y* variables explained by the model. Q^2^ reflected the predictive capacity of the model. The cumulative values of R^2^X, R^2^Y and Q^2^ varied from 0 to 1. Generally, the higher R^2^X (cum) and R^2^Y (cum), the more modeled variation used in the calculation. The higher the Q^2^ (cum) is, the better the predictability of the model is. Based on the variable importance (VIP) values (with a threshold of 1.0) obtained from the PLS-DA model, a number of metabolites responsible for the differentiation of the metabolic profiles were obtained. The compound identification was performed by comparing the mass fragments with the NIST standard mass spectral databases using a similarity of at least 80%.

### 3.7. Hierarchical Clustering and Pathway Enrichment Analysis

Hierarchical clustering was performed on the log-transformed normalized data. A small value (unity) was added to each normalized value to allow log transformation. The log-transformed data were median-centered per metabolite prior to clustering for better visualization. Pearson’s correlation was used for the similarity metric. The clustering was performed using the cluster program and visualized using the Mev software v4.9 [57]. A red/green color scheme was used in the heat maps of the metabolites. To determine which metabolic pathways are impacted by radiation exposure, the identified differential metabolites were mapped using the MetaboAnalyst 2.0 software [58]. For the analysis, the fruit fly (*Drosophila melanogaster*) pathway library, the hypergeometric test, and the out-degree centrality algorithms were employed. The software provided a fit coefficient (p) from the pathway enrichment analysis and an impact factor from the pathway topology analysis for each analyzed pathway.

## 4. Conclusions

In conclusion, the changes in somatic cell metabolites between the irradiated (20 and 40 Gy) and non-irradiated samples were investigated through a metabolomics approach in this study. The results showed that the metabolite levels were changed slightly in the 20-Gy samples but markedly altered in the 40-Gy samples compared with the non-irradiated samples. Marked disturbances in the metabolites in somatic cells can lead to biological malfunctions and thus diminish the competitiveness of the irradiated males, which will decrease the effectiveness of the SIT program. Previous studies have also shown that male beetles irradiated with 20 Gy exhibit a highly competitive mating capability and a longer mating period [17,18]. Thus, 20 Gy, a dose that results in partial sterilization and that can lead to high sterility, should be considered in the SIT for the control of *M*. *alternatus*. The findings of the current study will contribute to our knowledge of the mechanisms underlying the damage to somatic cells caused by gamma radiation to explore the SIT for improved pest control.

## Supplementary Files

Supplementary File 1 Supplementary Information (PDF, 578 KB)
