# Supplementary material for: Metabolic Profiling of Somatic Tissues from Monochamus alternatus (Coleoptera: Cerambycidae) Reveals Effects of Irradiation on Metabolism
Source: Int J Mol Sci. 2014 Jun 16;15(6):10806–20. doi: 10.3390/ijms150610806 (PMC4100182; doi:10.3390/ijms150610806)
Supplement: Supplementary File 1 — Supplementary Information (PDF, 578 KB) [file ijms-15-10806-s001.pdf]

Supplementary Information

Table S1. Result from pathway analysis of differential metabolites induced by 20 Gy irradiated males.

Pathway	Total	Expected	Hits	Raw p	−log(p)	Impact
Phenylalanine, tyrosine and tryptophan biosynthesis	4	0.071076	2	0.001753	6.3462	1
Phenylalanine metabolism	10	0.17769	2	0.012342	4.3948	0.69231
Aminoacyl-tRNA biosynthesis	67	1.1905	4	0.026433	3.6331	0
Ubiquinone and other terpenoid-quinone biosynthesis	3	0.053307	1	0.052416	2.9485	0
Alanine, aspartate and glutamate metabolism	23	0.40869	2	0.060525	2.8047	0.15203
Galactose metabolism	26	0.46199	2	0.07535	2.5856	0
Ascorbate and aldarate metabolism	6	0.10661	1	0.10223	2.2805	0
Histidine metabolism	7	0.12438	1	0.11828	2.1347	1
Beta-Alanine metabolism	13	0.231	1	0.20902	1.5653	0
Glycerolipid metabolism	16	0.2843	1	0.25101	1.3823	0
Propanoate metabolism	18	0.31984	1	0.27782	1.2808	0
Fructose and mannose metabolism	18	0.31984	1	0.27782	1.2808	0.15082
Pentose phosphate pathway	19	0.33761	1	0.29089	1.2348	0
Citrate cycle (TCA cycle)	20	0.35538	1	0.30373	1.1916	0.02566
Butanoate metabolism	21	0.37315	1	0.31635	1.1509	0
Pyruvate metabolism	24	0.42646	1	0.35293	1.0415	0
Inositol phosphate metabolism	24	0.42646	1	0.35293	1.0415	0.2043
Glutathione metabolism	26	0.46199	1	0.37628	0.97742	0
Tyrosine metabolism	30	0.53307	1	0.42061	0.86604	0.18795
Arginine and proline metabolism	37	0.65745	1	0.49113	0.71106	0
Purine metabolism	64	1.1372	1	0.69427	0.3649	0.01409

Table S2. Result from pathway analysis of differential metabolites induced by 40 Gy irradiated males.

Pathway	Total	Expected	Hits	Raw p	−log(p)	Impact
Aminoacyl-tRNA biosynthesis	67	2.7779	10	0.00022	8.4201	0
Alanine, aspartate and glutamate metabolism	23	0.9536	5	0.00186	6.2872	0.33784
Phenylalanine, tyrosine and tryptophan biosynthesis	4	0.16584	2	0.009555	4.6507	1
Galactose metabolism	26	1.078	4	0.019652	3.9296	0.40731
Nitrogen metabolism	7	0.29023	2	0.030884	3.4775	0
Citrate cycle (TCA cycle)	20	0.82922	3	0.046215	3.0745	0.09774
Phenylalanine metabolism	10	0.41461	2	0.061161	2.7942	0.69231
Valine, leucine and isoleucine biosynthesis	13	0.53899	2	0.098042	2.3224	0.33333
Ubiquinone and other terpenoid-quinone biosynthesis	3	0.12438	1	0.11941	2.1252	0
D-Glutamine and D-glutamate metabolism	5	0.20731	1	0.19116	1.6546	0
Arginine and proline metabolism	37	1.5341	3	0.19469	1.6363	0
Ascorbate and aldarate metabolism	6	0.24877	1	0.22486	1.4923	0
Cyanoamino acid metabolism	6	0.24877	1	0.22486	1.4923	0
Linoleic acid metabolism	6	0.24877	1	0.22486	1.4923	1
Histidine metabolism	7	0.29023	1	0.25719	1.3579	1
Glycine, serine and threonine metabolism	25	1.0365	2	0.27787	1.2806	0.32883
Glutathione metabolism	26	1.078	2	0.29363	1.2255	0
Methane metabolism	9	0.37315	1	0.31795	1.1459	0
Nicotinate and nicotinamide metabolism	9	0.37315	1	0.31795	1.1459	0
Tyrosine metabolism	30	1.2438	2	0.35608	1.0326	0.18795
Beta-Alanine metabolism	13	0.53899	1	0.42526	0.85505	0
Fatty acid biosynthesis	38	1.5755	2	0.4745	0.74549	0
Glyoxylate and dicarboxylate metabolism	16	0.66338	1	0.49475	0.70371	0
Starch and sucrose metabolism	17	0.70484	1	0.51603	0.66159	0.02799
Propanoate metabolism	18	0.7463	1	0.53644	0.6228	0
Fructose and mannose metabolism	18	0.7463	1	0.53644	0.6228	0.15082
Pentose phosphate pathway	19	0.78776	1	0.55601	0.58697	0
Butanoate metabolism	21	0.87068	1	0.59275	0.52298	0
Porphyrin and chlorophyll metabolism	23	0.9536	1	0.62653	0.46757	0
Tryptophan metabolism	23	0.9536	1	0.62653	0.46757	0.375
Pyruvate metabolism	24	0.99506	1	0.64237	0.44259	0
Inositol phosphate metabolism	24	0.99506	1	0.64237	0.44259	0.2043
Fatty acid elongation in mitochondria	27	1.1194	1	0.68607	0.37678	0
Amino sugar and nucleotide sugar metabolism	34	1.4097	1	0.76875	0.26299	0
Valine, leucine and isoleucine degradation	35	1.4511	1	0.77867	0.25017	0
Fatty acid metabolism	38	1.5755	1	0.806	0.21567	0
Pyrimidine metabolism	41	1.6999	1	0.83003	0.18629	0
Purine metabolism	64	2.6535	1	0.93919	0.062736	0

© 2014 by the authors; licensee MDPI, Basel, Switzerland. This article is an open access article distributed under the terms and conditions of the Creative Commons Attribution license (http://creativecommons.org/licenses/by/3.0/).
